# Do color enhancement algorithms improve the experience of color-deficient people? An empirical study based on smartphones

**DOI:** 10.3389/fnins.2024.1366541

**Published:** 2024-04-17

**Authors:** Yunhong Zhang, Yuelin Hu, Jun Tan, Ruiqing Ma, Feng Si, Yi Yang

**Affiliations:** ^1^Key Laboratory of Human Factors and Ergonomics for State Market Regulation, China National Institute of Standardization, Beijing, China; ^2^College of Computer Science and Technology (College of Data Science), Taiyuan University of Technology, Taiyuan, China

**Keywords:** color blindness, color weakness, smartphone, color-enhancing, user experience

## Abstract

Approximately 8% of the global population experiences color-vision deficiency. It is important to note that “color-vision deficiency” is distinct from “color blindness,” as used in this article, which refers to the difficulty in distinguishing certain shades of color. This study explores color enhancement algorithms based on the neural mechanisms of color blindness and color deficiency. The algorithms are then applied to smartphones to improve the user experience (UX) of color-enhancing features in different top-selling smartphone brands with different operating systems (OS). A color-enhancing application program was developed for individuals with color-vision deficiency and compared to two other mature color-enhancing programs found in top-selling smartphones with different mainstream operating systems. The study included both objective and subjective evaluations. The research materials covered three aspects: daily life, information visualization, and videos. Additionally, this research study examines various levels of color enhancement through three dimensions of subjective evaluation: color contrast, color naturalness, and color preference. The results indicate that all color-enhancing features are beneficial for individuals with color-vision deficiencies due to their strong color contrast. The users' color preference is closely linked to color naturalness. The application program preserves the naturalness of colors better than the other two color-enhancing features. The subjective evaluations show similar trends across different operating systems, with differences arising from the use of different color-enhancing algorithms. Therefore, different algorithms may result in different sizes of the color gamut.

## 1 Introduction

Over 80% of the information that human beings themselves acquire externally comes from vision. Color, being one of the most important visual parameters, is critical to information transmission. Color not only is an attraction to sensation but also helps people process and store pictures, enhancing memories (National Eye Institute, 2023). In daily life, most people suffering from color-vision deficiency (CVD) problems find it hard to distinguish specific colors, which is different from being “color-blind,” as given in this article. Color blindness means being incapable of distinguishing between certain shades of color. In addition, only a small number of people suffer from complete color blindness (0.00003%), in which everything is observed as shades of black and white (Wenjie et al., 2021). In other words, compared with color-blind individuals, individuals with CVD can distinguish specific colors in certain ways. However, color-enhancing features in top-selling brand smartphones cater to both color-blind and color-vision-deficient individuals by using filters that are effective for color-blindness. In other words, these filters enable color-blind individuals to differentiate and gather information by transforming colors that they are unable to or struggle to differentiate into colors that they can.

Due to the genetic mutation of the L or M cone in the retina, some people are congenitally red–green color-deficient. Red–green color deficiency can be divided into four categories: deuteranopia, protanopia, deuteranomaly, and protanomaly. Deuteranopia and protanopia individuals can match a test color with only two colored lights and are referred to as dichromats. Deuteranomaly and protanomaly individuals need a mixture of three colored lights to match a test color like in normal color-vision, but with different proportions, and are referred to as anomalous trichromats. According to Birch (2012), the rate of inherited red–green color deficiency in European Caucasians is approximately 8% in men and approximately 0.4% in women, of which approximately 1% includes deuteranopia, 1% protanopia, 1% protanomaly, and 5% deuteranomaly. The number of anomalous trichromats is much larger than that of dichromats. The color perception mechanisms for red–green color deficiency have been studied for a long time, including the color constancy mechanism (Foster, 2011), color naming (Nagy et al., 2014), and color discrimination (Boehm et al., 2021). Color image enhancement methods for dichromats have been extensively investigated (Kovalev and Petrou, 2005; Kuhn et al., 2008; Ching and Sabudin, 2010; Machado and Oliveira, 2010; Hassan and Paramesran, 2017; Hassan, 2019), while there are few studies (Mochizuki et al., 2008; Woo et al., 2018) about color image enhancement in anomalous trichromats. Accordingly, there are few studies on the evaluation of enhanced image quality in anomalous trichromats. The quality of enhanced images was only evaluated in terms of contrast and naturalness to demonstrate the effectiveness of the proposed recoloring algorithm (Machado et al., 2009).

These filters indeed work; however, for people who have color-vision deficiency problems, it seems like “excessive force.” Thus, an application program has been developed that aims to fit those with color-vision deficiency with easy-to-distinguish color information while ensuring maximum naturalness of color. Compared with the color-enhancing features in the market, this application program has a higher color gamut, which means fuller color, which makes green greener and blue bluer. It could effectively reduce negative influences on color naturalness by increasing the color contrast.

The article published by IEEE in 2018 proposed to provide a color blindness mode in different smartphone systems for color-blind consumers. However, the color blindness mode may sometimes improve customers' satisfaction rate, while in the majority of circumstances, the improvement in efficiency and the efficacy rate is not adequate; in addition, it can only perform a few specific tasks (Adler, 2021).

Most of the research studies on mobile phone color focus on color design, color psychology, and color correction algorithms, such as LMS, LAB, and Color-blind Filter Service (CBFS). The study of color enhancement characteristics from the perspective of ergonomics is still in its infancy. This study discusses the human–computer interface and color science from the perspective of ergonomics and pays more attention to the user experience of the color enhancement function in smartphones and the use of a variety of scenarios through the combination of experimental and interview methods. This research explores the application program and the effectiveness of different types of color-enhancing features, evaluating it with a combination of research and interviews. The research includes (1) the usefulness of the smartphone's color enhancement feature to color-vision deficiencies; compares (2) the color-enhancing features of three top-selling smartphones; and enhances (3) subjective sensation change to color-vision deficiencies under different color enhancement features.

## 2 Materials and methods

### 2.1 Research design

In this research, the application program for the color-vision deficiency group was developed and installed in the latest model of a top-selling smartphone and compared with other two mature color-enhancing features in different top-selling brand smartphones with different mainstream operating systems. The application program for the color-vision deficiency group is based on color adaptation algorithms for people with CVD people, with a focus on still images, yet they also apply to other media such as text and video. The methodology addresses the ability of color adaptation methods to preserve the perceptual learning of CVD people as much as possible. Thus, the following perceptual requirements must be satisfied to preserve perceptual learning: color naturalness, color consistency, and color contrast (Ribeiro and Gomes, 2020). The phone containing the research application program was named phone 1, and the other two were named phone 2 and phone 3. The independent variables of this research are the different color-enhancing features in the three mobile phones. In the subjective evaluation, dependent variables were subjective sensations and interviews from three different angles: color contrast, naturalness, and preference, and in the objective test, dependent variables were the completion time of color perception performance and the number of hits within the threshold.

### 2.2 Participants

There were a total of six participants in this research, three of them were color-blind, and the other three were color-vision-deficient. The age of all participants ranged from 18 to 40 years. Based on the distribution of the age level for smartphone usage: 96% of those aged 18 to 29 years use smartphones and 92% of those aged from 30 to 49 years use smartphones (Iqbal et al., 2018). Moreover, participants from age of 45 and above have an unalterable chance of suffering from macular disease, which may influence the results of color distinguishing (Andrew, 2022). Informed consent was obtained from each participant before the experiment, and upon completion, each participant received RMB¥ 200 as compensation. This study received ethical approval from the ethics committee of Taiyuan University of Technology.

### 2.3 Materials

**Figure 1:**
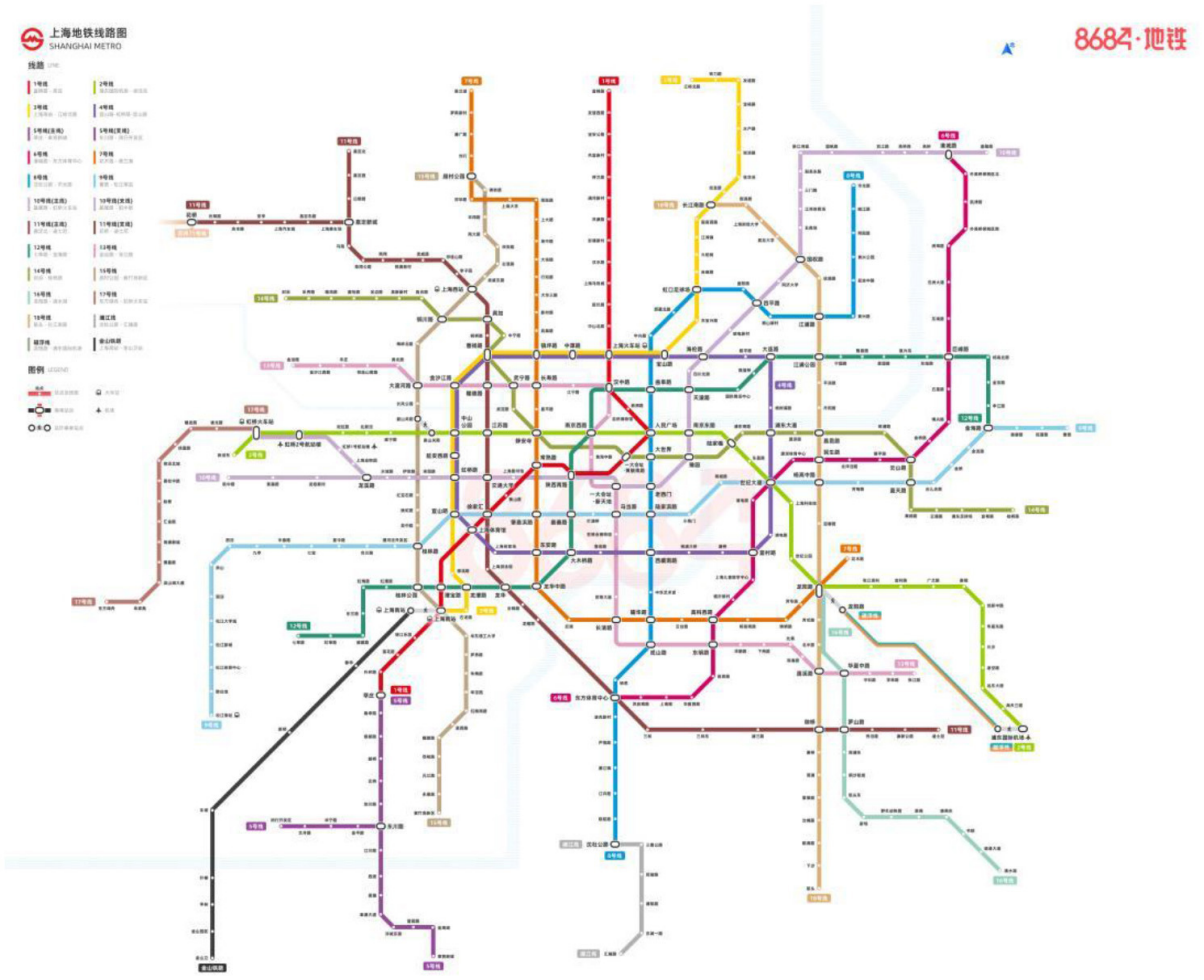
Example of visual information.

**Figure 2:**
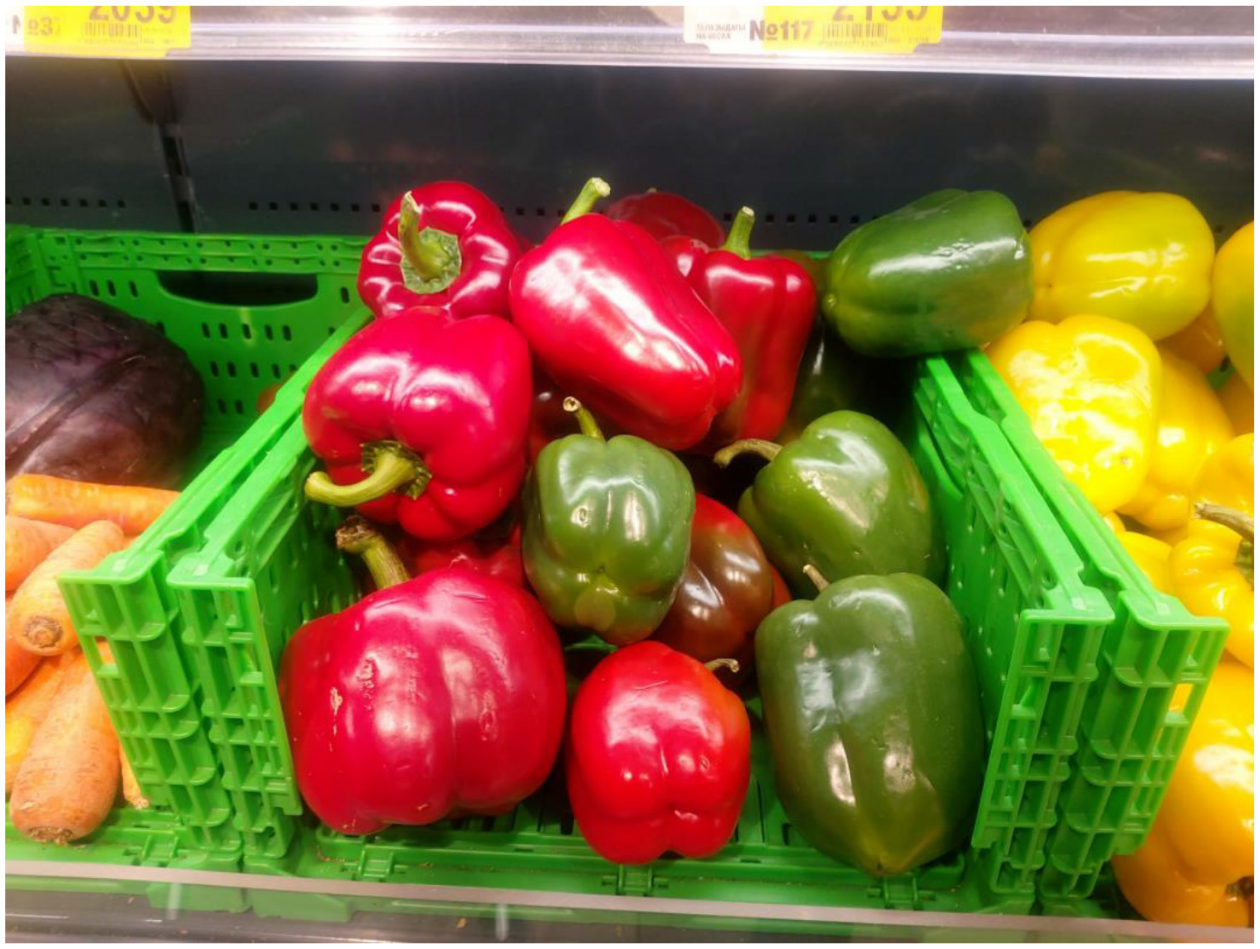
Example of daily life.

The research materials were chosen based on the diversity of circumstances related to the usage of smartphones; there are three categories: (1) daily life; (2) visual information; and (3) videos. Since the majority of color-vision deficiencies are red–green blindness [95% of color-vision deficiencies are red–green blindness (NHS, 2022)], most research materials were set to be focusing on colors that are hard to be distinguished in red–green blindness. There are five pictures and one video for evaluation. Here are some examples of subjective evaluation materials in Figures 1, 2.

### 2.4 Procedure

The research used Farnsworth–Munsel Dichotomous D-15 test products and Neitz Anomaloscope OT-II to make sure that color-enhancing feature filter on the smartphones used matched the participants' color-vision deficiency type.

The objective test application was only available in specific types of the operating systems in smartphones; in this research study, phone 1 and phone 2 were available for testing, and since mobile phone 3 was not available for the application program, it needed to be excluded. Before the beginning of the test, participants were asked to complete the built-in color-vision deficiency test on each phone; phone 1 and phone 2 automatically adjusted to the fittest color-enhancing filter based on their algorithm. Based on the filter adjusted, complete the objective test. This test is carried out to find one different block between a bunch of blocks of the same color. Thus, the completion time and the number of hits within the threshold are two test indicators to be considered. After completion of the test, the results were recorded.

Finally, the subjective evaluations were carried out, and different kinds of pictures and videos were provided to participants under the original, low, medium, and high levels (and phone 1 contained a color-blind level) of the color-vision deficiency mode on smartphones; all were provided to the participants in random order and participants were asked to rate them. Based on participants' subjective sensations, all pictures and videos under different modes were rated on different smartphones from score 1 to 5 and the results were recorded. In addition, the order of phones among all participants was balanced by the Latin square design.

After each group of pictures or videos had been rated, the experimenter would interview the participants, and the interview considered the following aspects: describing the color of the pictures, commenting on the pictures based on their subjective sensation, and commenting based on three different angles mentioned previously.

**Figure 3:**
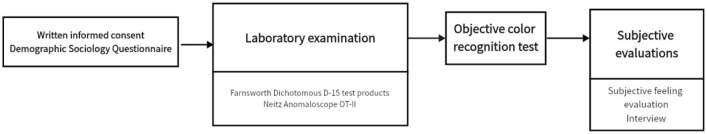
Experimental procedure.

The total experimental duration was approximately 1.5 h. The specific experimental procedure is shown in Figure 3.

## 3 Results

**Figure 4:**
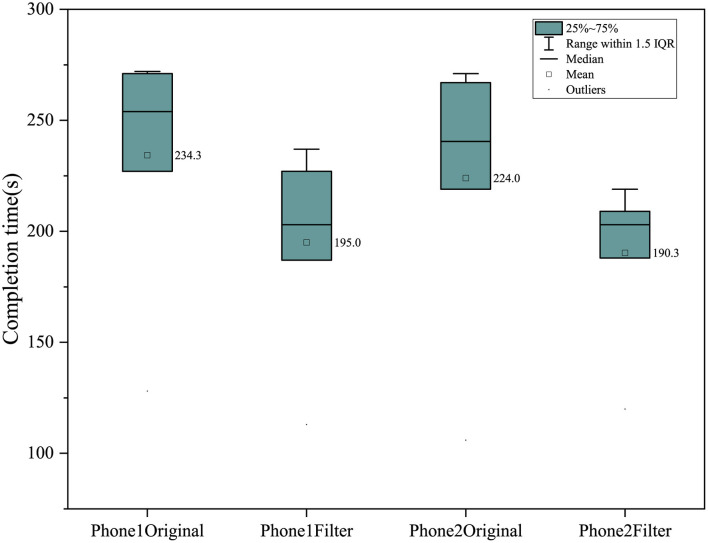
Completion time in different phones.

**Figure 5:**
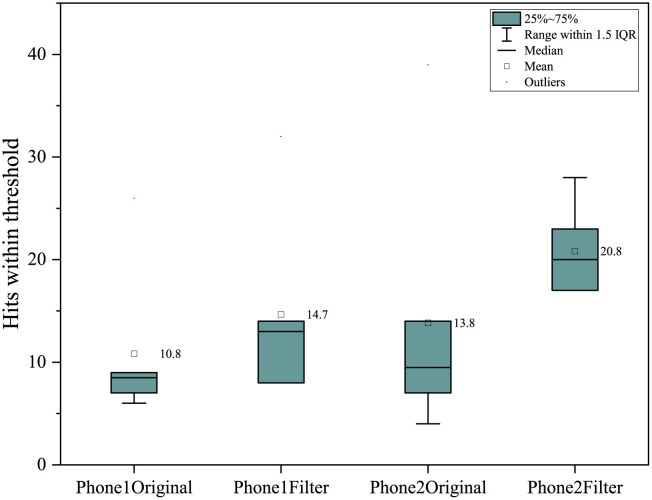
Number of hits within the threshold in different phones.

In the objective test, the completion time of phone 1 and phone 2 was significantly reduced, with an average reduction of 39.3 s after using color-enhancing mode in phone 1; the average decrease was 33.7 s after color-enhancing in phone 2 (see Figure 4). In addition, the number of hits within the hit threshold of mobile phone was increased after using the color-enhancing mode, with an average of 3.9 in phone 1; an average of 7 were added in phone 2 (as shown in Figure 5). The color-enhancing feature of phone 2 works better than that of phone 1 in both objective tasks on average.

During the subjective evaluations, to observe more clearly from three different angles, the data have been processed. All data are shown as the difference between the color-enhancing mode and the original mode. If the difference is positive, it means the color-enhancing mode works better than the original mode; if the difference is negative, it means the color-enhancing mode works worse than the original mode; if the difference is 0, it means the color-enhancing mode is the same as the original mode.

First, use all phones under all extents of the enhancing mode and analyze color contrast, naturalness, and preference results. Then, acquire the color contrast medians of the enhancing mode from all three mobile phones.

**Figure 6:**
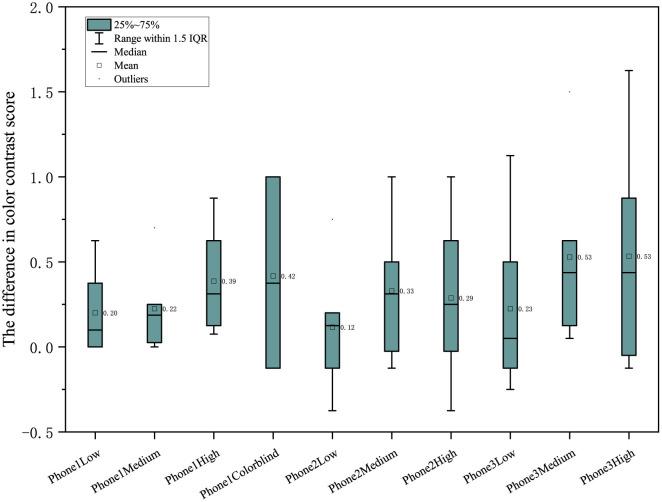
Comparison of color contrast under different color enhancing modes.

**Figure 7:**
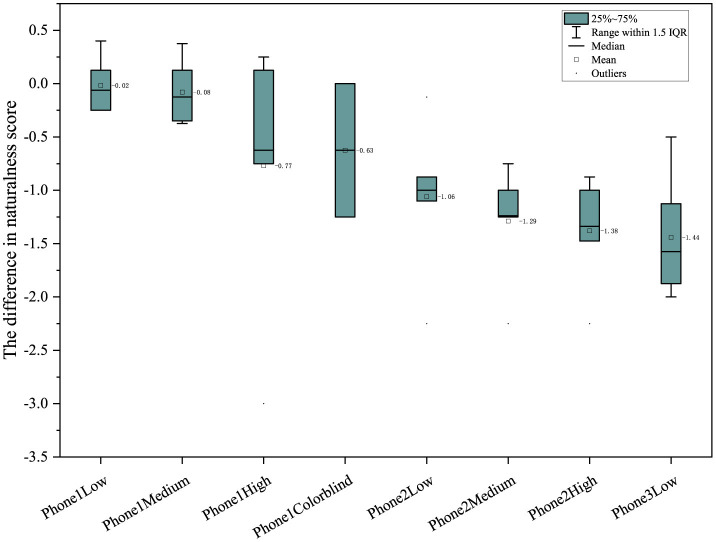
Comparison of naturalness under different color enhancing modes.

**Figure 8:**
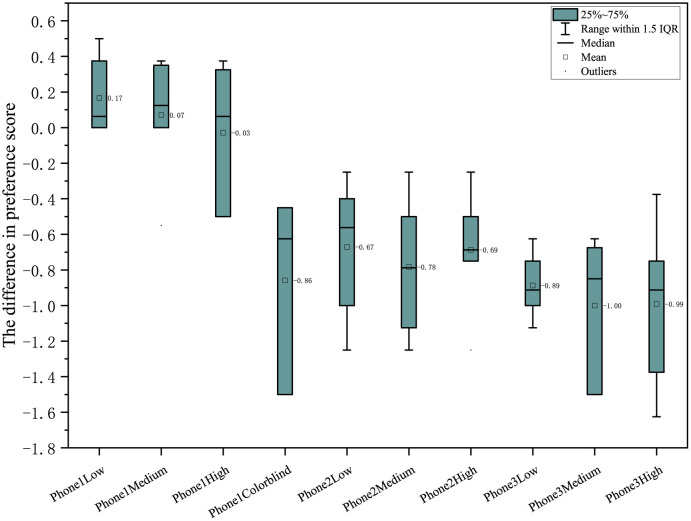
Comparison of preference under different color enhancing modes.

In color contrast, phone 1 has a difference >0 in general, while phone 2 and mobile phone 3 have the best performance under the medium enhancing mode (see Figure 6). In naturalness, phone 1 is generally better than phone 2 and phone 3, with all differences being positive (see Figure 7). Furthermore, in color preference, the score of phone 1 under different color-enhancing modes is better than that of phone 2 and phone 3. Generally, as the color- enhancing level increases, the preference shows a downward trend (see Figure 8).

After comparing the color contrast, naturalness, and preference of three smartphones under the different modes, count and analyze the overall color contrast, naturalness, and preference of three mobile phones. Based on the data, phone 3 performed significantly better than mobile phone 2 on color contrast (Table 1), also, phone 1 performed better than mobile phone 2 and mobile phone 2 performed better than mobile phone 3 on naturalness and preference (Tables 2, 3). Furthermore, naturalness and preference show a high level of positive correlation (Table 4), which means that, when naturalness increases, the preference increases with it, and color naturalness influences participants' preference for research materials.

**Table 1:** Comparison of color contrast in different smartphones.

**Brand**		**Mean deviation**	**Standard error**	**Significance**
Phone 3	Phone 2	0.185	0.072	0.063^*^

^*^indicates significant difference under 80% confidence interval.

**Table 2:** Comparison of naturalness in different smartphones.

**Brand**		**Mean deviation**	**Standard error**	**Significance**
Phone 1	Phone 2	0.901	0.317	0.047^*^
	Phone 3	1.269	0.289	0.012^*^
Phone 2	Phone 3	0.368	0.162	0.086^*^

^*^indicates significant difference under 80% confidence interval.

**Table 3:** Comparison of preference in different smartphones.

**Brand**		**Mean deviation**	**Standard error**	**Significance**
Phone 1	Phone 2	0.698	0.176	0.017^*^
	Phone 3	0.943	0.147	0.003^*^
Phone 2	Phone 3	0.246	0.12	0.11^*^

^*^indicates significant difference under 80% confidence interval.

**Table 4:** Relevance between naturalness and preference in different smartphones.

	**Phone 1 preference**	**Phone 2 preference**	**Phone 3 preference**
Phone 1 naturalness	0.889^*^	-	-
Phone 2 naturalness	-	0.551^*^	-
Phone 3 naturalness	-	-	0.895^*^

^*^indicates significant difference under 80% confidence interval.

## 4 Discussion

In the post-experiment interview, the satisfaction of the participants with the test mobile phone reached 100%. Based on the interviews with the participants after the research, all participants indicated that the improvement of contrast level in the color-enhancing mode helped them distinguish information: colors that were recognized as the same or similar can be discerned. In addition, color weakness participants had a strong sensation of color differences. Compared to their cognition during the evaluation of naturalness, color blindness participants only sensed color differences on mobile phone 2 and mobile phone 3; on the other hand, on mobile phone 1, color blindness participants indicated that the research material seemed to be roughly the same as the original mode under three enhancing modes (low, medium, and high), while participants would sense the difference from the original mode under the color blindness mode. It could prove that this application program has a good performance on color contrast, at the same time it can keep a high quality on color naturalness and color preference. Most color-vision deficiency people do not need to change color to help them distinguish easily; when the color becomes fuller, they can recognize it.

As for color preference, according to previous research studies, the low efficiency and satisfaction of color-vision deficiency users correlate with digital products because of ignoring their own color perception (Xia et al., 2012). A similar conclusion was also reflected in this research. The objective and subjective evaluations prove this perspective, and there will be more preference when closer to nature and closer to the sensation of the real world is experienced for participants. The users' color preference is highly positively related to color naturalness. The more natural the color, that is, the color is similar to their own color perception, the higher the user's color preference score.

The results of this study also further confirm that the enhancement features of the application for people with color-vision deficits have a positive effect on their recognition and use, which is consistent with the observation of some studies (Iqbal et al., 2018). There are a large number of people with color blindness, and they face many inconveniences due to their inability to correctly identify colors in daily life. However, due to the medical level and technology, there is no effective treatment plan at present. The society has not given much attention to the color blind group, and people lack the understanding of the perspectives of color blindness in life. In the field of design, the barrier-free design for the color-blind group mainly focuses on the design of color-blind glasses, color-blind recognizable color barrier-free color matching systems, and traffic lights, among others. These designs can solve the problem of color-blind people in recognizing color information in some cases, but they cannot be widely used in daily life, and there are certain limitations. Therefore, by evaluating the user experience of the color enhancement function in smartphones, more attention could be paid to the use of various features, which is conducive to promoting the convenience of using smart products for people with color-vision defects.

This research discusses user experience (UX), meaning a person's perceptions and responses resulting from the use and anticipated use of a product (ISO, 2022). Thus, in this research, the objective evaluation could reflect the effectiveness of parameters related to color-enhancing features' performance. The subjective evaluations and interviews could directly reflect how participants feel and their opinions about each task and highlight the strength and weakness of these three different color-enhancing features. It could help these color-enhancing features to be upgraded in the future.

## 5 Conclusion

In conclusion, the application program could help CVD people distinguish colors and, moreover, keep a high color naturalness level and high color preference. With the combination of research and interviews of all participants, the color-enhancing mode has a positive effect on color-vision deficiencies; by increasing the level of contrast, color-vision deficiencies would distinguish color-related information more clearly. This concept is based on human beings' visual systems being more sensitive to high contrast levels. In addition, color-vision deficient people have their own senses of color from life; for instance, although the color reflected in their brains is different from that of those who are not suffering color-vision deficiencies, they still use the same name of the color as regular people. It can be reflected by when the experimenter increased the contrast level of the research phones: participants would state that they have never seen such red or green in their daily life, which ultimately caused a decline in preference. Previous research stated that, because of the ignorance of color sensation from color-vision deficiencies, the satisfaction rate and effective interaction rate of color-vision deficiencies have been kept at a low level (National Eye Institute, 2022).

Therefore, future research on color enhancement should focus on how to maintain the most naturalness while increasing the contrast level. Only then are both the color-related information distinguishing ability and satisfaction rate considered, which meets the concept of user experience (UX) research: designs based on human needs and mutual coordination between humans and machines.

## Data availability statement

The original contributions presented in the study are included in the article/supplementary material, further inquiries can be directed to the corresponding author.

## Ethics statement

The studies involving humans were approved by Taiyuan University of Technology. The studies were conducted in accordance with the local legislation and institutional requirements. The participants provided their written informed consent to participate in this study. Written informed consent was obtained from the individual(s) for the publication of any potentially identifiable images or data included in this article.

## Author contributions

YZ: Writing—review & editing, Writing—original draft, Visualization, Validation, Project administration, Methodology. YH: Writing—review & editing, Writing—original draft. JT: Writing—review & editing, Writing—original draft. RM: Writing—review & editing, Writing—original draft. FS: Writing—review & editing, Writing—original draft. YY: Writing—review & editing, Writing—original draft.
